# Supplementary material for: An ambruticin-sensing complex modulates Myxococcus xanthus development and mediates myxobacterial interspecies communication
Source: Nat Commun. 2020 Nov 4;11:5563. doi: 10.1038/s41467-020-19384-7 (PMC7643160; doi:10.1038/s41467-020-19384-7)
Supplement: Supplementary file 1 — Supplementary Information [file 41467_2020_19384_MOESM1_ESM.pdf]

Supplementary Information

An ambruticin-sensing complex modulates *Myxococcus xanthus* development and mediates myxobacterial interspecies communication

Francisco Javier Marcos Torres[1,+], Carsten Volz[1,+] and Rolf Müller[1,2]*

[1] Department of Microbial Natural Products (MINS), Helmholtz Institute for Pharmaceutical Research Saarland (HIPS), Helmholtz Centre for Infection Research (HZI), Campus E8 1, 66123 Saarbrücken, Germany

[2] German Center for Infection Research (DZIF), Braunschweig 38124, Germany

[+] These authors contributed equally to this work.

* To whom correspondence should be addressed: Prof. Rolf Müller; Tel: +49 681 98806-3000; Email: rolf.mueller@helmholtz-hips.de

- **Supplementary Figures**
- **Supplementary Tables**

Supplementary Figures

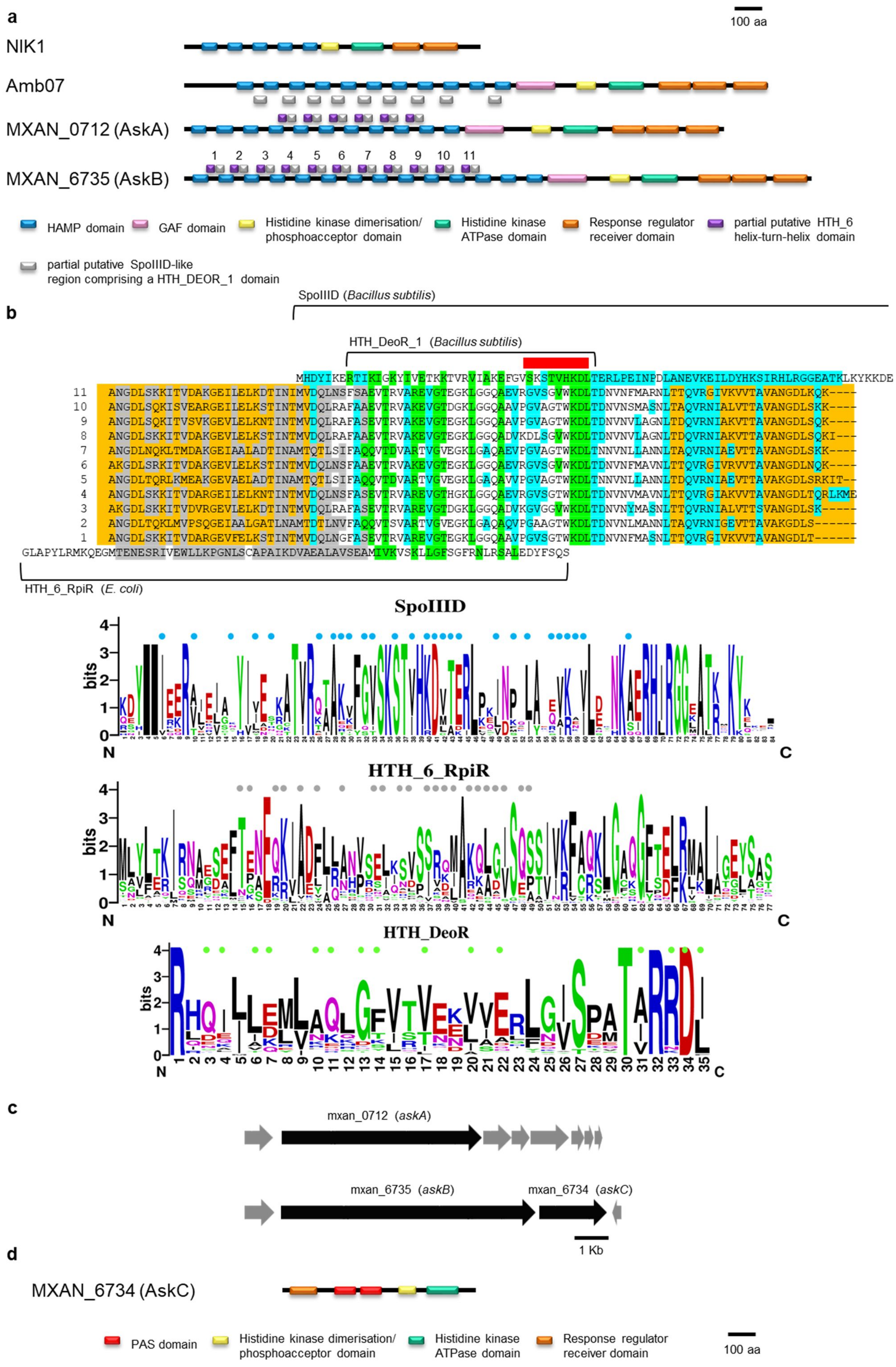

a
100 aa
NIK1
Amb07
MXAN_0712 (AskA)
1 2 3 4 5 6 7 8 9 10 11
MXAN_6735 (AskB)
HAMP domain
GAF domain
Histidine kinase dimerisation/ phosphoacceptor domain
Histidine kinase ATPase domain
Response regulator receiver domain
partial putative HTH_6 helix-turn-helix domain
partial putative SpoIIID-like region comprising a HTH_DEOR_1 domain
b
SpoIIID (Bacillus subtilis)
HTH_DeoR_1 (Bacillus subtilis)
MHDYIKERTIKIGKYIVETKKTVRVIAKEFGVSKSTVHKDLTERLPEINPDLANEVKEILDYHKSIRHLRGGEATKLKYKKDE
11 ANGDLSKKITVDAKGEILELKDTINIMVDQLNSFSAEVTRVAREVGTEGKLGGQAEVRGVSGVWKDLTDNVNFMARNLTTQVRGIVKVVTAVANGDLKQK----
10 ANGDLSQKISVEARGEILELKSTINTMVDQLRAFAAEVTRVAKEVGTDGKLGGQAAVPGVAGTWKDLTDNVNSMASNLTAQVRNIALVTTAVANGDLSKK----
9 ANGDLSQKITVSVKGEVLELKNTINTMVDQLRAFASEVTRVAKEVGTEGKLGGQAAVPGVAGTWKDLTDNVNVLAGNLTDQVRNIAKVTTAVANGDLSQK----
8 ANGDLSKKITVDAKGEVLELKSTINTMVDQLRAFAAEVTRVAKEVGTEGKLGGQADVKDLSGVWKDLTDNVNVLAGNLTDQVRNIAKVTTAVANGDLSQKI---
7 ANGDLNQKLTMDAKGEIAALADTINAMTQTLSIFAQQVTDVARTVGVEGKLGAQAEVPGVAGTWKDLTNNVNLLANNLTAQVRNIAEVTTAVANGDLSKK----
6 AKGDLSRKITVDAKGEVLELKSTINTMVDQLNSFAAEVTRVAKEVGTEGKLGGQAEVRGVSGVWKDLTDNVNFMAVNLTTQVRGIVRVVTAVANGDLNQK----
5 ANGDLTQRLKMEAKGEVAELADTINAMTQTLSIFAQQVTDVARTVGVEGKLGAQAVVPGVAGTWKDLTNNVNLLANNLTDQVRNIAEVTTAVAKGDLSRKIT--
4 ANGDLSKKITVDARGEILELKNTINTMVDQLNSFASEVTRVAREVGTHGKLGGQAEVRGVSGTWKDLTDNVNVMAVNLTTQVRGIAKVVTAVANGDLTQRLKME
3 AKGDLSKKITVDVRGEVLELKDTINTMVDQLRAFASEVTRVAREVGTDGKLGGQADVKGVGGVWKDLTDNVNYMASNLTTQVRNIALVTTSVANGDLSK-----
2 ANGDLTQKLMVPSQGEIAALGATLNAMTDTLNVFAQQVTSVARTVGVEGKLGAQAQVPGAAGTWKDLTDNVNLMANNLTAQVRNIGEVTTAVAKGDLS------
1 ANGDLSKKITVDARGEVFELKSTINTMVDQLNGFASEVTRVAREVGTEGKLGGQAAVPGVSGTWKDLTDNVNFMASNLTTQVRGIVKVVTAVANGDLT------
GLAPYLRMKQEGMTENESRIVEWLLKPGNLSCAPAIKDVAEALAVSEAMIVKVSKLLGFSGFRNLRSALEDYFSQS
HTH_6_RpiR (E. coli)
SpoIIID
bits
N
C
HTH_6_RpiR
bits
N
C
HTH_DeoR
bits
N
C
c
mxan_0712 (askA)
mxan_6735 (askB)
mxan_6734 (askC)
1 Kb
d
MXAN_6734 (AskC)
PAS domain
Histidine kinase dimerisation/ phosphoacceptor domain
Histidine kinase ATPase domain
Response regulator receiver domain
100 aa

Fig. S1 Domain analysis and genetic organization of the ambruticin sensor kinases AskABC. **a** Domain architecture of the ambruticin sensitivity determinant NIK1 from *C. albicans*, Amb07 from *S. cellulosum* So ce10, and MXAN_0712 (AskA) and MXAN_6735 (AskB) from *M. xanthus*. AskAB exhibit a 63.5% and a 52.9% amino acid identity with Amb07 of strain Soce10, respectively. HTH_6_RpiR domains (Pfam accession PF01418) and SpoIIID-like regions (Pfam PF12116) comprising HTH_DeoR_1 domains (Pfam PF08220) were searched in all shown proteins. Partial SpoIIID-like regions comprising a HTH_DeoR_1 domain could be found in Amb07, AskA and AskB. Partial HTH_6_RpiR domains could only be found in AskA and AskB. In AskA and AskB, partial HTH_6_RpiR domains and HTH_DeoR_1 always occurred as pairs in a fixed orientation (HTH_6_RpiR followed by HTH_DeoR_1 with respect to the N-terminus of AskB). In AskB, these pairs have been marked with numbers (1-11). **b** Alignment of HTH_6_RpiR and SpoIIID/HTH_DeoR_1 with 11 regions of AskB comprising pairs of partial HTH_6_RpiR/HTH_DeoR_1. HTH domains of RpiR (*E.coli*) and SpoIIID (HTH_DeoR_1; *Bacillus subtilis*) are given above and below the sequences of the 11 regions. Residues within the 11 sequences in accordance with the sequence logos (bottom) of HTH_DeoR_1, HTH_6, and SpoIIID are highlighted in green, grey and blue, respectively. The respective positions are also highlighted in the sequence logos themselves (colored circles). Residues highlighted in orange color should be part of the predicted HAMP domains. A red box above amino acid residues highlights the DNA recognition helix of SpoIIID as proposed earlier[1]. Sequence logos were generated using Weblogo[2,3]. **c** Genetic environment of MXAN_0712 (AskA), MXAN_6735 (AskB) and MXAN_6734. All genes analyzed in this study are highlighted in black. **d** Domain architecture of MXAN_6734 (AskC).

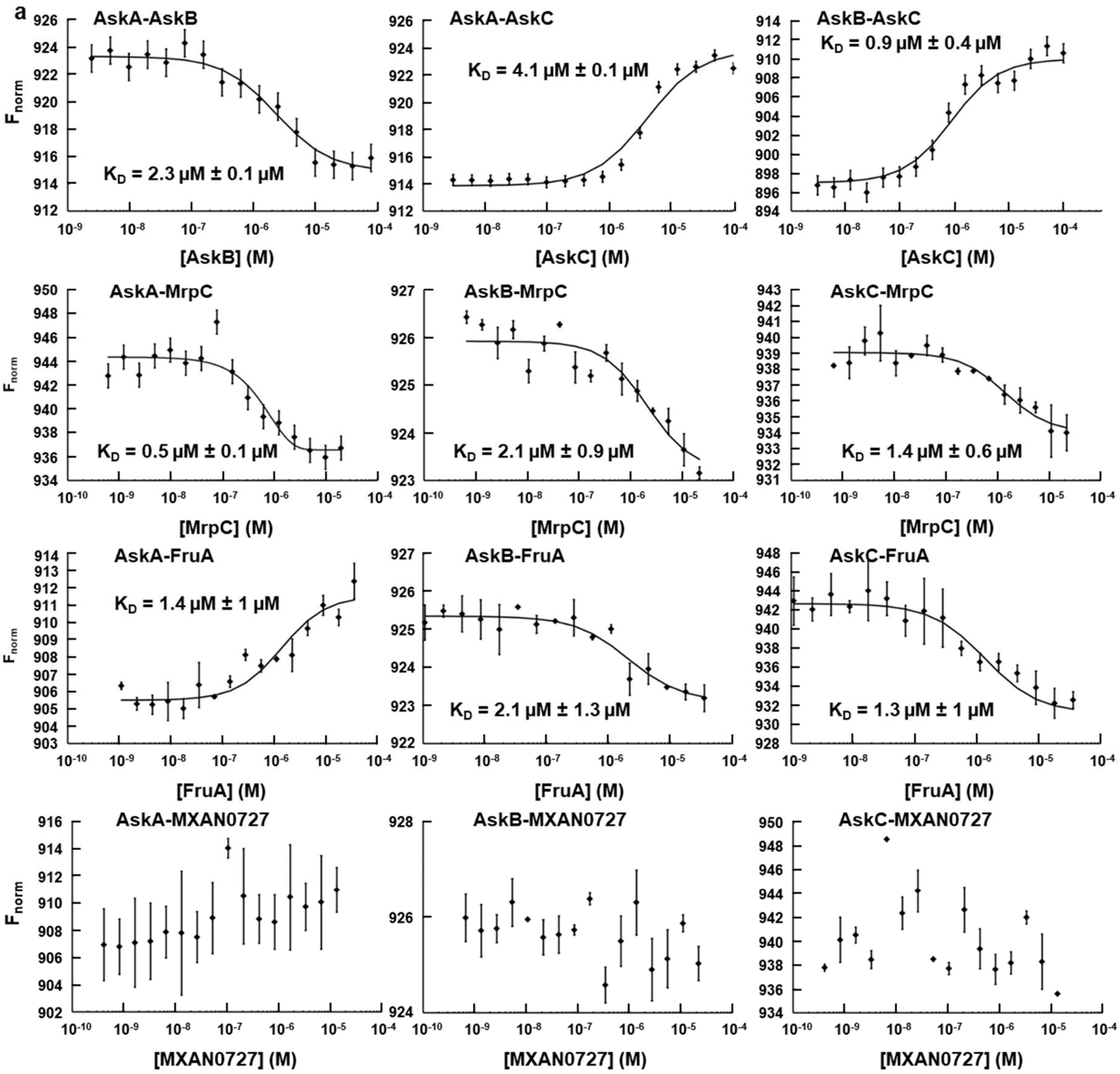

b

$$F(C_T) = F_A + \frac{F_{AT} - F_A}{2C_A}\left(C_T + C_A + K_D - \sqrt{(C_T + C_A + K_D)2 - 4C_T C_A}\right)$$

Fig. S2 Pairwise interaction of AskA, AskB, AskC, MrpC and FruA as investigated by MST analysis. AskA, AskB, and AskC specifically interact with both, MrpC and FruA. MXAN_0727 was used as a negative control and exhibited no interaction with all tested proteins. As a second negative control, bovine serum albumin (BSA) was chosen also exhibiting no interaction (not shown). **a** Dose response curves of NT-647-labeled and C-terminally-His-tagged proteins AskA-NT647, AskB-NT647, AskC-NT647 and non-labeled, AskA, AskB, AskC, MrpC, FruA and MXAN_0727 are shown. Fnorm on Y-axis: normalized fluorescence in ‰. Concentration of the unlabeled ligand is shown on the x-axis (M). Experiments were performed in triplicates on a Monolith™ NT.115 instrument (NanoTemper). Response values were averaged and plotted against the concentration of the ligands. For K_D determination, values were fit using MO.Affinity Analysis software (NanoTemper, for further details refer to Method's section). Obtained K_D values are depicted above or below each dose response curve. **b** Quadratic equation used within the MO.Affinity Analysis software (refer to Methods section). F_A = response value of unbound NT-647-labeled proteins. F_{AT} = response value of the complex of NT-647-labeled and unlabeled molecules. C_A = concentration of NT-647-labeled protein. C_T = Concentration of the unlabeled ligand.

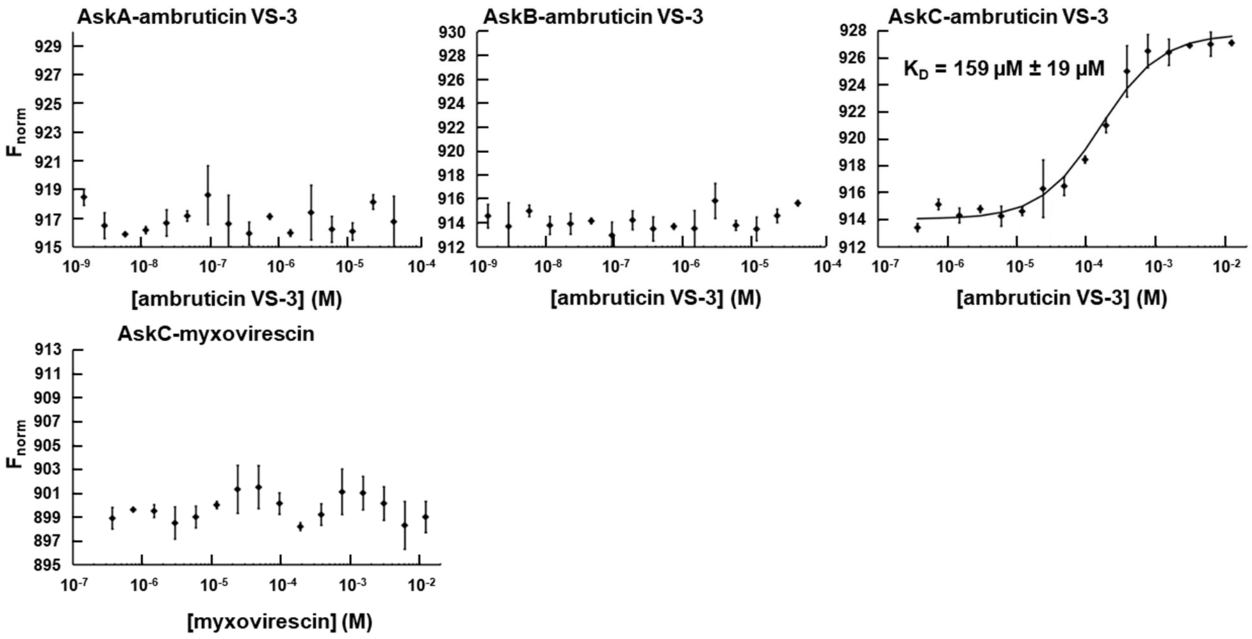

Fig. S3 Interaction of AskA, AskB and AskC with ambruticin VS-3 as investigated by MST analysis. Dose response curves of NT-647-labeled C-terminally-His-tagged proteins AskA-NT647, AskB-NT647, AskC-NT647 and ambruticin VS-3 or myxovirescin A are shown. Fnorm on Y-axis: normalized fluorescence in ‰. Concentration of the unlabeled ligand is shown on the x-axis (M). Experiments were performed in triplicates on a Monolith™ NT.115 instrument (NanoTemper). Response values were averaged and plotted against the concentration of the ligands. For K_D determination, values were fit using MO.Affinity Analysis software (NanoTemper, for further details refer to Method's section). Obtained K_D values are depicted above or below each dose response curve.

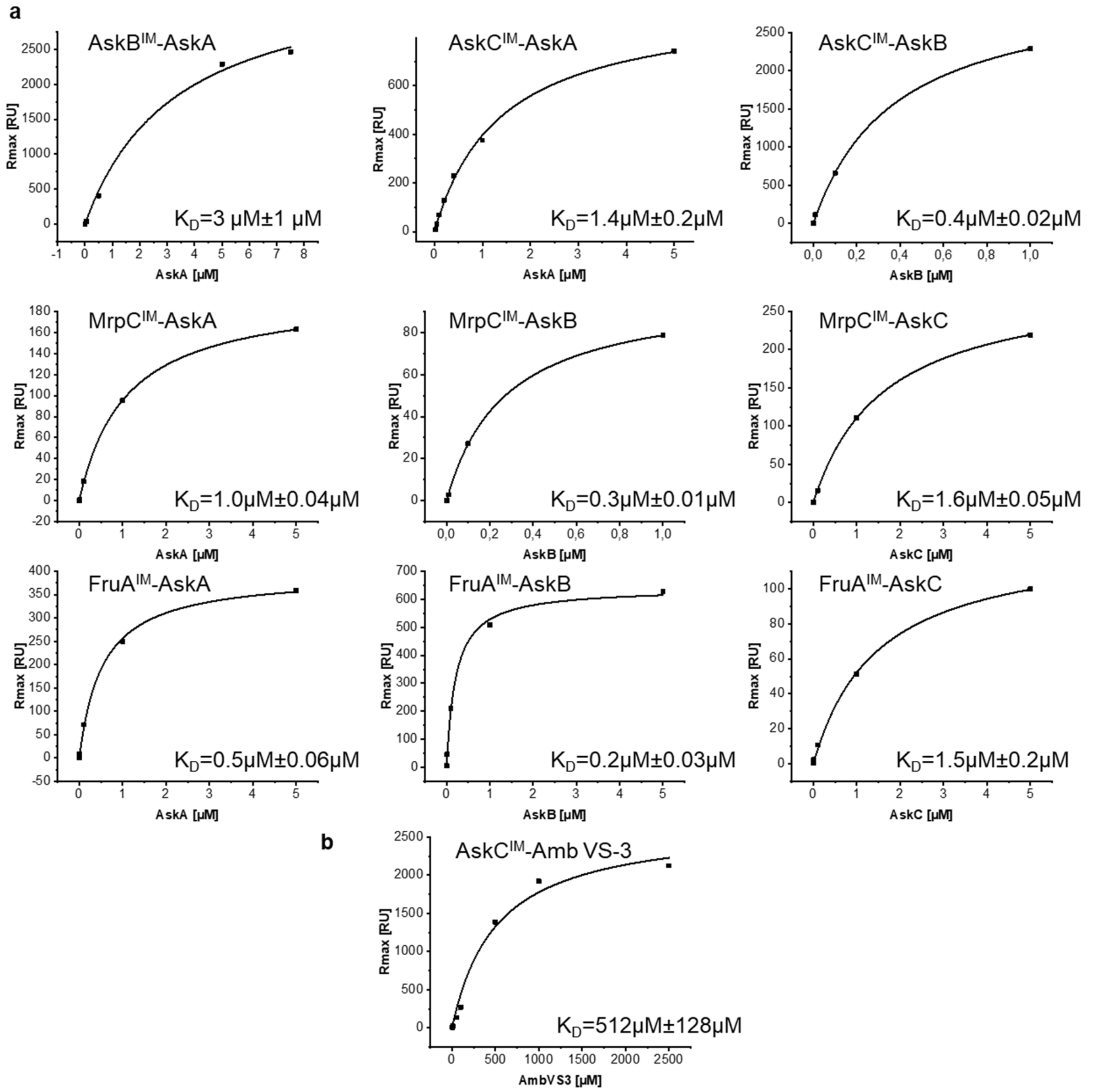

Fig. S4 Pairwise interaction of AskA, AskB, AskC, MrpC, FruA and AskC-ambruticin VS-3 as investigated by SPR equilibrium analysis. Representative plots of Rmax values of each tested pairs of proteins exhibiting specific interaction are shown. Pairings are indicated above each blot. The immobilized ligand of each pair is indicated by the affix “IM”. Rmax on Y-axis: maximum response in response units (RU). Concentration of the analyte protein is shown on the x-axis (µM). Curves depict fitted functions obtained using Biacore X100 Evaluation software Version 2.0.1 (GE Healthcare). Calculated K_D values are given in each plot. Each experiment was performed in triplicates. See Methods section for further details. **a** AskA, AskB, and AskC specifically interact with each other kinase as well as with both, MrpC and FruA. MXAN_0727 and BSA were used as a negative control and exhibited no interaction with all tested proteins (not shown). **b** AskC specifically interacts with ambruticin VS-3. No interaction could be observed using any other protein including MrpC (not shown).

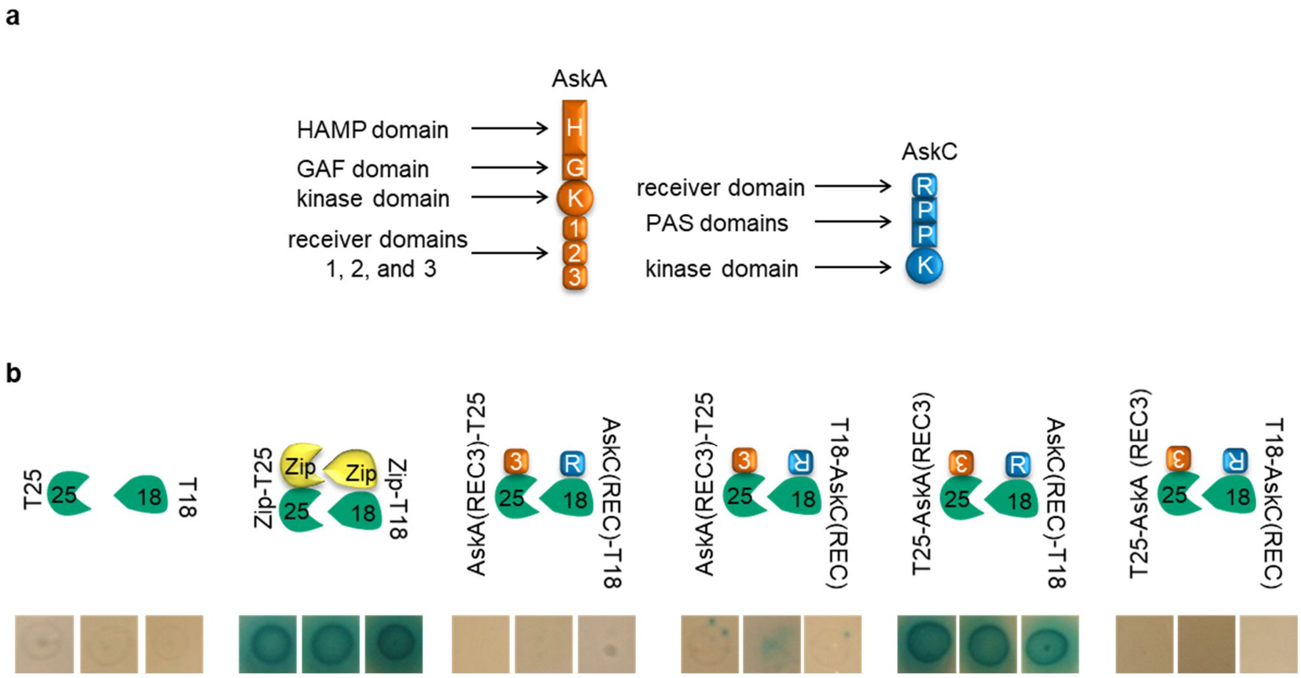

Fig. S5 Interaction of the AskA REC3 domain and the REC domain of AskC. **a** Scheme of the domain organization of AskA and AskC. **b** BACTH assay on M63 medium using the REC3 domain of AskA and the receiver domain REC of AskC as fusion proteins with T25 and T18, respectively. Each set of three replicates is accompanied by a graphic representation of their respective proteins and tested orientations (see in the Methods section). A positive interaction results in development of blue color in X-gal containing plates.

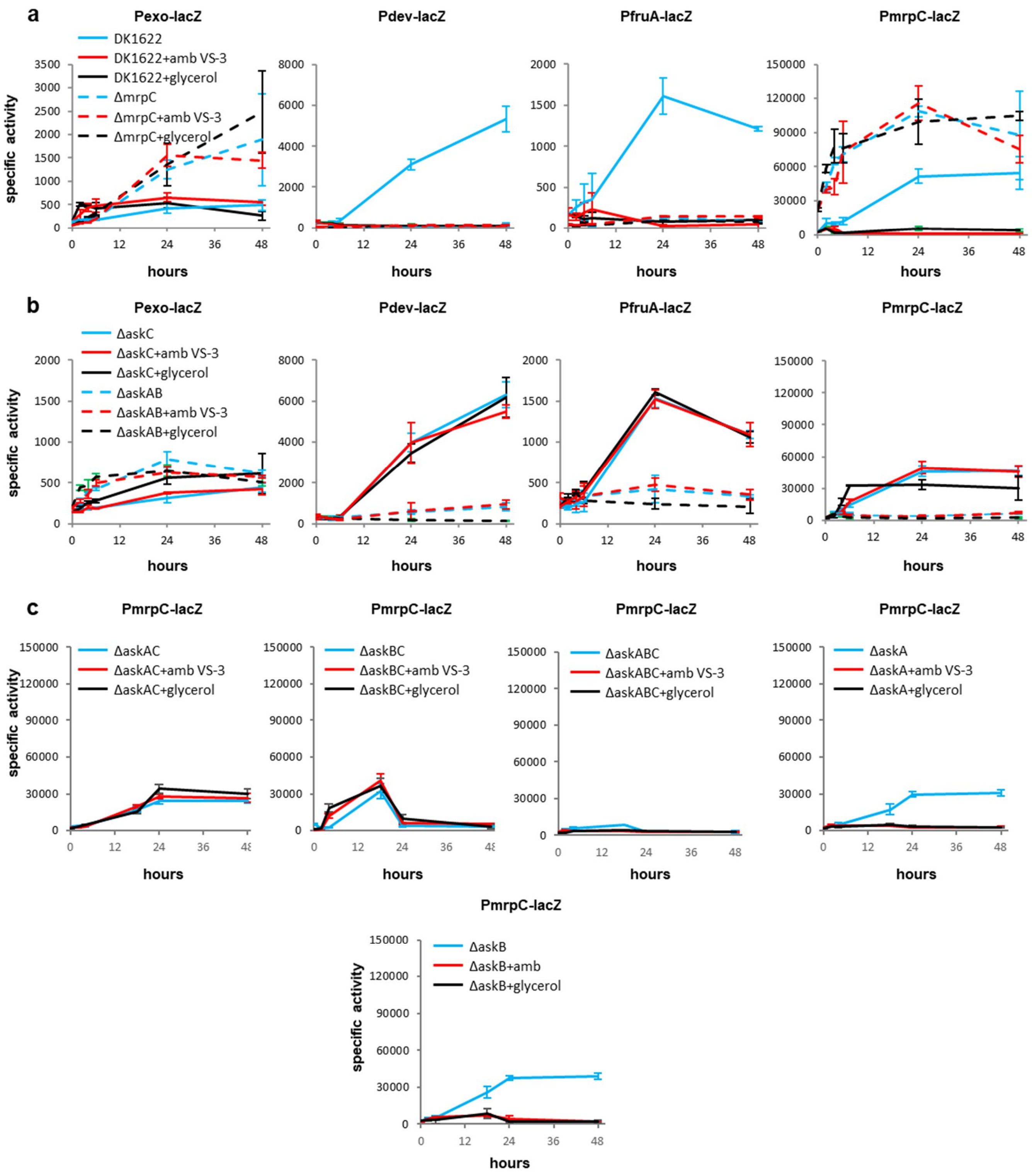

Fig. S6 Effects of ambruticin VS-3 and glycerol on selected promoters of the *M. xanthus* developmental gene regulatory network. **a** β-galactosidase specific activity of *exo*, *dev*, *fruA*, and *mrpC* promoter-*lacZ* fusions Pexo-lacZ, Pdev-lacZ, PfruA-lacZ and PmrpC-lacZ during FB formation on CF starvation medium in absence (blue lines) or in presence of 60 nm ambruticin VS-3 (red lines) or 300 mM glycerol (black lines) in *M. xanthus* DK1622 (continuous lines) and *M. xanthus* ΔmrpC (discontinuous lines). **b** β-galactosidase specific activity of *exo*, *dev*, *fruA*, and *mrpC* promoter-*lacZ* fusions during FB formation on CF starvation medium in absence (blue lines) or in presence of 60 nm ambruticin VS-3 (red lines) or 300 mM glycerol (black lines) in *M. xanthus* ΔaskC (continuous lines) and *M. xanthus* ΔaskAB (discontinuous lines). **c** ß-galactosidase specific activity of *mrpC* promoter-*lacZ* fusions during FB formation on CF starvation medium in absence (blue lines) or in presence of 60 nm ambruticin VS-3 (red lines) or 300 mM glycerol (black lines) in *M.xanthus* ΔaskAC, *M. xanthus* ΔBC and *M. xanthus* ΔaskABC. β-galactosidase

specific activity is expressed as picomoles of 4-methylumbelliferone released per minute per milligram of protein. Error bars represent the standard deviation of three independent replicates.

Supplementary Tables

Table S1. Bacterial strains used in this study.

bacterial strains	genotype/phenotype[a]	reference or source
E. coli		[4]
DH10B	F$^-$ mcrA Δ(*mrr-hsd*RMS-*mcr*BC) Φ80d*lac*ZΔM15 Δ*lac*X74 *end*A1 *rec*A1 *deo*R Δ(*ara,leu*)7697 *ara*D139 *gal*U *gal*K *nup*G *rps*L λ$^-$	
E. coli BL21 (DE3)	*fhuA2* [*lon*] *ompT gal* (λ DE3) [*dcm*] Δ*hsdS* λ DE3 = λ sBamHIo *Δ*EcoRI-B *int*::(*lacI*::P*lacUV5*::T7 gene 1) i21 *Δnin5* T1 phage-resistant version of BL21(DE3)	NEB strain catalogue no. C2527
E. coli BHT101	F$^-$ *cya-99 araD139 galE15 galK16 rpsL1* (Str^r) *hsdR2 mcrA1 mcrB1*	[5]
S. cellulosum		[6]
Soce10	Wild type strain	
M. xanthus		[7]
DK1622	Wild type strain	
ΔaskA	DK1622-Δ*askA*, markerless deletion mutant of gene *askA* (MXAN_0712)	This study
ΔaskB	DK1622-Δ*askB*, markerless deletion mutant of gene *askB* (MXAN_6735)	This study
ΔaskAB	DK1622-Δ*askAB*, markerless double deletion mutant of genes *askA* (MXAN_0712) and *askB* (MXAN_6735)	This study
ΔaskBC	DK1622-Δ*askBC*, markerless double deletion mutant of genes *askB* (MXAN_6735) and *askC* (MXAN_6734)	This study
ΔaskAC	DK1622-Δ*askAC*, markerless double deletion mutant of genes *askA* (MXAN_0712) and *askC* (MXAN_6734)	This study
ΔaskABC	DK1622-Δ*askABC*, markerless triple deletion mutant of genes *askA* (MXAN_0712), *askB* (MXAN_6735) and *askC* (MXAN_6734)	This study
Δ0715	DK1622-Δ*0715*, markerless deletion mutant of gene MXAN_0715	This study
ΔaskC	DK1622-Δ*askC*, markerless deletion mutant of gene *askC* (MXAN_6734)	This study
ΔmrpC	DK1622-Δ*mrpC*, markerless deletion mutant of gene *mrpC* (MXAN_5125)	This study
DK1622-Pexo	DK1622 with genomic integration of promoter test vector pCKßgal_P*exo* via the attB/P-site, KanR	This study
DK1622-Pdev	DK1622 with genomic integration of promoter test vector pCKßgal_P*dev* via the attB/P-site, KanR	This study
DK1622-PfruA	DK1622 with genomic integration of promoter test vector pCKßgal_P*fruA* via the attB/P-site, KanR	This study
DK1622-PmrpC	DK1622 with genomic integration of promoter test vector pCKßgal_P*mrpC* via the attB/P-site, KanR	This study
ΔaskAB-Pexo	Δ*askAB* with genomic integration of promoter test vector pCKßgal_P*exo* via the attB/P-site, KanR	This study
ΔaskAB-Pdev	Δ*askAB* with genomic integration of promoter test vector pCKßgal_P*dev* via the attB/P-site, KanR	This study
ΔaskAB-PfruA	Δ*askAB* with genomic integration of promoter test vector pCKßgal_P*fruA* via the attB/P-site, KanR	This study
ΔaskAB-PmrpC	Δ*askAB* with genomic integration of promoter test vector pCKßgal_P*mrpC* via the attB/P-site, KanR	This study
ΔaskC-Pexo	Δ*askC* with genomic integration of promoter test vector pCKßgal_P*exo* via the attB/P-site, KanR	This study
ΔaskC-Pdev	Δ*askC* with genomic integration of promoter test vector pCKßgal_P*dev* via the attB/P-site, KanR	This study
ΔaskC-PfruA	Δ*askC* with genomic integration of promoter test vector pCKßgal_P*fruA* via the attB/P-site, KanR	This study
ΔaskC-PmrpC	Δ*askC* with genomic integration of promoter test vector pCKßgal_P*mrpC* via the attB/P-site, KanR	This study
ΔmrpC-Pexo	Δ*mrpC* with genomic integration of promoter test vector pCKßgal_P*exo* via the attB/P-site, KanR	This study
ΔmrpC-Pdev	Δ*mrpC* with genomic integration of promoter test vector pCKßgal_P*dev* via the attB/P-site, KanR	This study
ΔmrpC-PfruA	Δ*mrpC* with genomic integration of promoter test vector pCKßgal_P*fruA* via the attB/P-site, KanR	This study
ΔmrpC-PmrpC	Δ*mrpC* with genomic integration of promoter test vector pCKßgal_P*mrpC* via the attB/P-site, KanR	This study
ΔaskAC-PmrpC	Δ*askAC* with genomic integration of promoter test vector pCKßgal_P*mrpC* via the attB/P-site, KanR	This study

ΔaskBC-PmrpC	Δ*askBC* with genomic integration of promoter test vector pCKßgal_P*mrpC* via the attB/P-site, KanR	This study
ΔaskABC-PmrpC	Δ*askABC* with genomic integration of promoter test vector pCKßgal_P*mrpC* via the attB/P-site, KanR	This study

[a]KanR indicates resistance to kanamycin.

Table S2. Plasmids used in this study.

plasmids	relevant features or genes	reference or source
pSWU41	*sacB*, *nptII*, KanR	[8]
pSWU41-ΔaskA	ΔaskA, *sacB*, KanR	This study
pSWU41-ΔaskB	ΔaskB, *sacB*, *nptII*, KanR	This study
pSWU41-Δ0715	Δ0715, *sacB*, *nptII*, KanR	This study
pSWU41-ΔaskC	ΔaskC, *sacB*, *nptII*, KanR	This study
pSWU41-ΔmrpC	ΔmrpC, *sacB*, *nptII*, KanR	This study
pCKßgal_att_ter	*lacZ*, *nptII*, *ble*, KanR, ZeoR	[9]
pCKßgal_Pexo	pCKßgal_att_ter; Pexo-*lacZ*, *nptII*, *ble*, KanR, ZeoR	This study
pCKßgal_Pdev	pCKßgal_att_ter; Pdev-*lacZ*, *nptII*, *ble*, KanR, ZeoR	This study
pCKßgal_PfruA	pCKßgal_att_ter; PfruA-*lacZ*, *nptII*, *ble*, KanR, ZeoR	This study
pCKßgal_PmrpC	pCKßgal_att_ter; PmrpC-*lacZ*, *nptII*, *ble*, KanR, ZeoR	This study
pE-SUMO3kan	His6-SUMO-tag, *nptII*, KanR	Life Sensors
pE-SUMO3kan-mrpC	*SUMO-mrpC*, *nptII*, KanR	This study
pE-SUMO3kan-fruA	*SUMO-fruA*, *nptII*, KanR	This study
pE-SUMO3kan0727	SUMO-mxan_0727, *nptII*, KanR	This study
pE-SUMO3kan-askA	SUMO-*askA*, *nptII*, KanR	This study
pE-SUMO3kan-askB	SUMO-*askB*, *nptII*, KanR	This study
pET28C(+)	His(x6), *nptII*, KanR	Novagen
pET28C-askA	askA-His(x6), *nptII*, KanR	This study
pET28C-askB	askB-His(x6), *nptII*, KanR	This study
pET28C-askC	askC-His(x6), *nptII*, KanR	This study
pKT25	T25, *nptII*, KanR	Euromedex
pKT25-askA(REC3)	*T25-askA*(REC3), *nptII*, KanR	This study
pKNT25	T25, *nptII*, KanR	Euromedex
pKNT25-askA(REC3)	*askA*(REC3), *nptII*, KanR	This study
pUT18	T18, *bla*, AmpR	Euromedex
pUT18-askC(REC)	*askC*(REC)-T18, *bla*, AmpR	This study
pUT18C	T18, *bla*, AmpR	Euromedex

Table S3. Oligonucleotides used in this study

oligonucleotide	purpose	sequence (5’→3’)[a]
US712F	Amplification of upstream region of mxan_0712 (*askA*) to obtain strain ΔaskA	GCTCTAGACACGCAGGTCCAGGTG
US712R	Amplification of upstream region of mxan_0712 (*askA*) to obtain strain ΔaskA	**GGGCCCCGTCATCC**GGCCATATGAGTTCCTGGTC
DS712F	Amplification of downstream region of mxan_0712 (*askA*) to obtain strain ΔaskA	**GACCAGGAACTCATATGGCC**GGATGACGGGGCCC
DS712R	Amplification of downstream region of mxan_0712 (*askA*) to obtain strain ΔaskA	GCGGATCCGGCAGCAGCGGGTC
US6735F	Amplification of upstream region of mxan_6735 (*askB*) to obtain strain ΔaskB	GCTCTAGACAGCGTCGCGTCCAG
US6735R	Amplification of upstream region of mxan_6735 (*askB*) to obtain strain ΔaskB	**GAGGTTTCTCGCGTGGAC**GCTTGATTCGCTGAGTGATG
DS6735F	Amplification of downstream region of mxan_6735 (*askB*) to obtain strain ΔaskB	**CATCACTCAGCGAATCAAGC**GTCCACGCGAGAAACCTC
DS6735R	Amplification of downstream region of mxan_6735 (*askB*) to obtain strain ΔaskB	GCGGATCCGCACTTCGGC
US6734F	Amplification of upstream region of mxan_6734 (*askC*) to obtain strain ΔaskC	GCTCTAGAGGACCTCGAGCATGGAGAG
US6734R	Amplification of upstream region of mxan_6734 (*askC*) to obtain strain ΔaskC	**CACCTTGAGAACAGGTGGGA**AACTGAGCGCGTCCCAC
DS6734F	Amplification of downstream region of mxan_6734 (*askC*) to obtain strain ΔaskC	**GTGGGACGCGCTCAGTT**TCCCACCTGTTCTCAAGGTG
DS6734R	Amplification of downstream region of mxan_6734 (*askC*) to obtain strain ΔaskC	GCGGATCCGCAAGGAGGGCCTGGAG
USmrpCF	Amplification of upstream region of mxan_5125 (*mrpC*) to obtain strain ΔmrpC	GAGGGATCCCGGCGTCAAACGCATCAAG
USmrpCR	Amplification of upstream region of mxan_5125 (*mrpC*) to obtain strain ΔmrpC	**GCGCGCGGACTACTTCTC**ACCGTGCATGGCATAACTC
DSmrpCF	Amplification of downstream region of mxan_5125 (*mrpC*) to obtain strain ΔmrpC	**GAGTTATGCCATGCACGGT**GAGAAGTAGTCCGCGCGC
DSmrpCR	Amplification of downstream region of mxan_5125 (*mrpC*) to obtain strain ΔmrpC	CGTCTAGAAGCTGAGCGCCTTCGCG
Sumo0727F	Amplification of gene mxan_0727 for construction of plasmid pE-SUMO3kan-0727	GAGGGATCCATGGAATCGGAACGCATCC
Sumo0727R	Amplification of gene mxan_0727 for construction of plasmid pE-SUMO3kan-0727	CCGGTACCAGCTAGCCCCGCGGCGAC
LacZexoF	Amplification of promoter region of mxan _3225 (*exo* promoter) for construction of plasmid pCKßgal_Pexo	GAGGGATCCGCGCGAACTTCAGTCCAAG
LacZexoR	Amplification of promoter region of mxan _3225 (*exo* promoter) for construction of plasmid pCKßgal_Pexo	AATATCTGTACACAGCCCTCCCTCAAAATCG
LacZdevF	Amplification of promoter region of mxan _7265 (*dev* promoter) for construction of plasmid pCKßgal_Pdev	GCGGATCCGCCTTTGGGGCAATACAGG
LacZdevR	Amplification of promoter region of mxan _7265 (*dev* promoter) for construction of plasmid pCKßgal_Pdev	GCTGTACAACGCCTCGCAGCATCA
LacZfruAF	Amplification of promoter region of mxan _3117 (*fruA* promoter) for construction of plasmid pCKßgal_PfruA	GCGGATCCTTGACAGACAGCGCCG
LacZfruAR	Amplification of promoter region of mxan _3117 (*fruA* promoter) for construction of plasmid pCKßgal_PfruA	GCTGTACATGCGAAGGCCCCC
LacZmrpCF	Amplification of promoter region of mxan_5125 (*mrpC* promoter) for construction of plasmid pCKßgal_PmrpC	GCGGATCCGATCCTTGGAATCAGCCG
LacZmrpCR	Amplification of promoter region of mxan_5125 (*mrpC* promoter) for construction of plasmid pCKßgal_PmrpC	GCTGTACATGGCATAACTCCTGGGAAG
Sumo0712-F	Amplification of gene mxan_0712 (*askA)* for construction of plasmid pE-SUMO3kan-askA	GAGGGATCCATGGCCACGAAGAAGGTTTC
Sumo0712-R	Amplification of gene mxan_0712 (*askA*) for construction of plasmid pE-SUMO3kan-askA	GCGGTACCTCATCCGGAGACCTCCGTG
Sumo6735-F	Amplification of gene mxan_6735 (*askB*) for construction of plasmid pE-SUMO3kan-askB	GAGGGATCCGTGGACGACACCAAGGTTCCC

Sumo6735-R	Amplification of gene mxan_6735 (*askB)* for construction of plasmid pE-SUMO3kan-askB	GCGGTACCTCAAGCCGTCACCCACAGACGG
0712-C-F	Amplification of gene mxan_0712 (*askA*) for construction of plasmid pET28C-askA	ATTCTCATGATGGCCACGAAGAAGGTTTC
0712-C-R	Amplification of gene mxan_0712 (*askA*) for construction of plasmid pET28C-askA	GCTAACTCGAGTCCGGAGACCTCCGTG
6735C-HisF	Amplification of gene mxan_6735 (*askB*) for construction of plasmid pET28C-askB	GCACATGTGTGGACGACACCAAGGTTCCC
6735C-HisR	Amplification of gene mxan_6735 (*askB*) for construction of plasmid pET28C-askB	CGGCGGCCGCAGCCGTCACCCACAGACGG
SumomrpCF	Amplification of gene mxan_5125 (*mrpC*) for construction of plasmid pE-SUMO3kan-mrpC	GCGGATCCATGCACGGTTTCAACCGC
SumomrpCR	Amplification of gene mxan_5125 (*mrpC*) for construction of plasmid pE-SUMO3kan-mrpC	GCCCTCGAGCTACTTCTCCTTGCCGGCG
6734CHisF	Amplification of gene mxan_6734 (*askC*) for construction of plasmid pET28C-askC	GCACATGTTGGTGGGACCGGGGCA
6734CHisR	Amplification of gene mxan_6734 (*askC*) for construction of plasmid pET28C-askC	CGGCGGCCGCGTTGGCGGAGCGCG
SumofruAF	Amplification of gene mxan_3117 (*fruA*) for construction of plasmid pE-SUMO3kan-fruA	GCGGATCCATGGCAACCAATCAAGCAG
SumofruAR	Amplification of gene mxan_3117 (*fruA*) for construction of plasmid pE-SUMO3kan-fruA	GCGCTCGAGCTAGAGGTCCGGCGGC
BTH712RR3F	Amplification of the receiver domain Rec3 of mxan _0712 (*askA*) for construction of plasmids pKT25-askA(REC3) and pKNT25-askA(REC3)	GCTCTAGAGGTGAACCGCAAGGTGCT
BTH712R	Amplification of the receiver domain Rec3 of mxan _0712 (*askA*) for construction of plasmids pKT25-askA(REC3) and pKNT25-askA(REC3)	GCGGATCCGATCCGGAGACCTCCGTG
BTH6734F	Amplification of the receiver domain of mxan _6734 (*askC*) for construction of plasmids pUT18-askC(REC) and pUT18C-askC(REC)	GCTCTAGAGGTGGGACCGGGGCA
BTH6734RRR	Amplification of the receiver domain of mxan _6734 (*askC*) for construction of plasmids pUT18-askC(REC) and pUT18C-askC(REC)	GCGGATCCGACTGCCGGAGGAGCACC
BiotPmrpCF	5'-biotinylated primer for amplification of the *mrpC* promoter region for DNA-protein pull-down	[Biot]-GATCCTTGGAATCAGCCG
BiotPmrpCR	5'-biotinylated primer for amplification of the *mrpC* promoter region for DNA-protein pull-down	[Biot]-TGGCATAACTCCTGGGAAG
HEXPmrpCF	5' HEX-labelled primer for amplification of the *mrpC* promoter region for EMSA analysis (PmrpC)	[HEX]-GATCCTTGGAATCAGCCG
HEXPmrpCR	5' HEX-labelled primer for amplification of the *mrpC* promoter region for EMSA analysis (PmrpC)	[HEX]-TGGCATAACTCCTGGGAAG
Cy5-P0552F	5' Cy5-labelled primer for amplification of the mxan_0552 promoter region (Pmxan_0552) for EMSA analysis	[Cy5]-GCTCTTTCTTAGCTCGTGGGAATTG
P0552R	primer for amplification of the mxan_0552 promoter region (Pmxan_0552) for EMSA analysis	CAATTCCCACGAGCTAAGAAAGAGC

[a] Underlined nucleotides depict the restriction site sequences used in cloning. Homology regions for overlap extension PCR are marked in bold. 5'-modifications are indicated as follows: biotinylation [Biot], fluorescent labels [HEX] or [Cy5].

1. Himes, P., McBryant, S. J. & Kroos, L. Two regions of Bacillus subtilis transcription factor SpoIIID allow a monomer to bind DNA. *Journal of Bacteriology* **192,** 1596–1606; 10.1128/JB.01506-09 (2010).
2. Crooks, G. E., Hon, G., Chandonia, J.-M. & Brenner, S. E. WebLogo: a sequence logo generator. *Genome research* **14,** 1188–1190; 10.1101/gr.849004 (2004).

3. Schneider, T. D. & Stephens, R. M. Sequence logos: a new way to display consensus sequences. *Nucleic acids research* **18,** 6097–6100; 10.1093/nar/18.20.6097 (1990).
4. Grant, S. G., Jessee, J., Bloom, F. R. & Hanahan, D. Differential plasmid rescue from transgenic mouse DNAs into Escherichia coli methylation-restriction mutants. *Proceedings of the National Academy of Sciences of the United States of America* **87,** 4645–4649; 10.1073/pnas.87.12.4645 (1990).
5. Karimova, G., Pidoux, J., Ullmann, A. & Ladant, D. A bacterial two-hybrid system based on a reconstituted signal transduction pathway. *Proceedings of the National Academy of Sciences of the United States of America* **95,** 5752–5756 (1998).
6. Höfle, G., Steinmetz, H., Gerth, K. & Reichenbach, H. Antibiotics from gliding bacteria, XLIV. Ambruticins VS. New members of the antifungal ambruticin family fromSorangium cellulosum. *Liebigs Ann. Chem.* **1991,** 941–945; 10.1002/jlac.1991199101161 (1991).
7. Kaiser, D. Social gliding is correlated with the presence of pili in Myxococcus xanthus. *Proceedings of the National Academy of Sciences of the United States of America* **76,** 5952–5956; 10.1073/pnas.76.11.5952 (1979).
8. Wu, S. S. & Kaiser, D. Markerless deletions of pil genes in Myxococcus xanthus generated by counterselection with the Bacillus subtilis sacB gene. *Journal of Bacteriology* **178,** 5817–5821 (1996).
9. Volz, C., Kegler, C. & Müller, R. Enhancer binding proteins act as hetero-oligomers and link secondary metabolite production to myxococcal development, motility, and predation. *Chemistry & biology* **19,** 1447–1459; 10.1016/j.chembiol.2012.09.010 (2012).
